# Treatment of a Fractured Tooth

**Published:** 1875-09

**Authors:** Theodore G. Lewis


### ARTICLE V.

### *Treatment of a Fractured Tooth.*

BY THEODORE G. LEWIS, D. D. S.

Read before the Eighth District Dental Society, Buffalo, N. Y. May, 1873.

This case of a fracture of a tooth is reported as presenting features and perhaps suggestions that may on some future occasion be of service, or, at least serve as a hint in the treatment of an analogous case.

The occurrence of two cases exactly similar is quite improbable, as are many operations within the scope of dentistry. In filling teeth, for instance, no two cavities are precisely alike, yet the principle which enables us to prepare and properly fill one cavity, is applicable to other cavities to a certain extent; so in the following, the principle involved may enable one to treat successfully a similar case of fracture.

Mr. P. sustained injuries by being thrown from a sleigh sometime in January, 1875. Among the injuries was one to the left superior central incisor.

An examination some two months subsequent to the accident found the gums much inflamed immediately over the injured tooth, with a profuse discharge of pus. The crown of the tooth very loose and capable of being moved something like three-sixteenths of an inch either way from its normal position. By placing the fingers over the root of the tooth and impairing motion to it, crepitation was readily detected, indicating that the labial plate of the alveolus directly over the tooth was fractured; the palatine portion of the socket remaining intact.

An incision was made over the tooth sufficiently large to remove a small portion of alveolar plate which was found displaced, and the fact was then revealed that the tooth itself was fractured diagonally toward the palatine surface, the crown being entirely separated from the root and only retained in position by attachment of the soft parts to the lingual side of the tooth.

The fracture was situated about five thirty-seconds of an inch above the margin of the gum, and was from front, downwards and backwards, at an angle of about seventy degrees, as near as could be ascertained.

The pulp of the tooth previous to the accident had died or been devitalized, and after having the operation called rhizodontrypy performed upon it by one operator, had been successfully treated and the root filled with gold by another. The condition of the tooth previous to coming to my notice was as follows: a large right-approximal gold filling; pulp destroyed and nerve canal filled; but the opening made when rhizodontrypy was performed, left open. The fracture occurred at the immediate point where the orifice had been drilled, it evidently being the weakest part of the tooth.

It was obvious that the presence of pus was not due to a diseased condition of the tooth or consequent upon the fracture, but from the small pieces of detached alveolus that acted as an exciting cause of inflammation. After the removal of these, an application of a saturated solution of iodine in creosote was made and repeated every third day. Three applications sufficed to restore the soft parts to a nearly healthy condition.

The patient being a public lecturer, did not wish to be deprived of the tooth, as it would greatly impair his enunciation, and as pivoting an artificial crown to the root was impracticable, in consequence of it being broken so high up, it was at length determined to *bolt* the natural crown to the already separated root.

To successfully do this, it became necessary to devise some mechanical means to firmly hold the crown in its place,—as it would be impossible to drill a hole in the crown in its loose condition,—and in an exact line with the nerve canal in the root; besides, it was desirable to exclude from the joint the chips that would be made by the drill. To accomplish this, an impression of five anterior teeth, including the defective one, was taken, and a band of hard rubber made, about three-sixteenths of an inch wide, and encircling the five teeth above mentioned. This held the fractured tooth firmly in position while drilling.

The drills used were twist drills,—such as are used by workers in metal,—of three sizes, viz., Nos. 55, 53 and 44. Stubb's wire guage, cemented into handles by means of shellac. The burring engine not being used in this case as it was important to *feel* the progress of the work.

Drill No. 55 was first used and carried through the crown and to about five-sixteenths of an inch above the fracture, then followed by drill No. 53 through the *crown* of the tooth only. Drill No. 44 was then used to enlarge the orifice to within one-eighth of an inch of the fracture. This gave a shoulder for the bolt-head to draw up against. The hole in the root was now ready to be tapped out, or screw cut, which was done with a tap of suitable size.

The bolt or screw was made of Stubb's steel wire. Having used a steel screw on previous occasions for securing artificial crowns to roots, and found it to answer admirably, it was preferred in this case to one of gold, by reason of its greater strength, and, as I believe, answering a better purpose than gold.

A piece of the steel wire No. 42 was taken, sufficiently long to admit of its being handled conveniently, and one end filed till it was reduced to three sixty-fourths of an inch in diameter, and seven-sixteenths of an inch long. A thread was then cut on it five-sixteenths long. It was then inserted in the tooth before being cut from the steel, to ascertain if it fitted perfectly. After removal from the tooth, the screw was cut from the steel wire, leaving sufficient metal above the shoulder to form a head one sixteenth of an inch high, which after being nicked for propuls on by a small screw driver, completed the bolt. It was now placed in position in the tooth, and screwed into the root till the crown was brought firmly against the fractured surface. A gold filling over the head of the screw completes the operation.

The result proved very satisfactory, and the crown being so securely bolted to its root, was equally as firm as any of the teeth in the vicinity.

Its permanency may be questioned, not as to the security of the crown to the root, for there is scarcely a possibility of its ever being dislocated, but pathologically. Still, the conditions remaining the same as at the time of its completion, it will certainly be as permanent as an ordinary artificial pivot tooth; and it seems probable that the fracture being so high up and protected by the gums, that there is less liability of the joint being disintegrated by the action of the fluids of the mouth, than are pivoted roots with the joint between crown and root, immediately at the margin of the gum.—*Dental Advertiser.*

---

